# Retraction: Elevated microRNA-145 inhibits the development of oral squamous cell carcinoma through inactivating ERK/MAPK signaling pathway by down-regulating HOXA1

**DOI:** 10.1042/BSR-2018-2214_RET

**Published:** 2022-11-10

**Keywords:** Extracellular-signal-regulated kinase/mitogen activated protein kinase, Homeobox A1, MicroRNA-145, Oral squamous cell carcinoma

This Retraction follows an Expression of Concern relating to this article previously published by Portland Press. This article is being retracted from *Bioscience Reports* at the request of the Editor-in-Chief and the Editorial Board following receipt of a notification from a reader, alerting the Editorial Board to similar regions that appear in both the miR-145 mimic and siHOXA1 panels of figure 4C, as well as concerns regarding the western blots of figures 6 and 7. The authors have been contacted with regards to the retraction and have not responded to the Journal's queries or concerns raised. Given the extent of the issues raised, the Editorial Board stand by the decision to retract the article.

